# Biofilm-Related Infections in Healthcare: Moving towards New Horizons

**DOI:** 10.3390/microorganisms12040784

**Published:** 2024-04-12

**Authors:** Enea Gino Di Domenico, Alessandra Oliva, María Guembe

**Affiliations:** 1 Department of Biology and Biotechnology “C. Darwin”, Sapienza University of Rome, 00185 Rome, Italy; enea.didomenico@uniroma1.it; 2 Department of Public Health and Infectious Diseases, Sapienza University of Rome, 00185 Rome, Italy; alessandra.oliva@uniroma1.it; 3 Department of Clinical Microbiology and Infectious Diseases, Hospital General Universitario Gregorio Marañón, 28007 Madrid, Spain; 4 Instituto de Investigación Sanitaria Gregorio Marañón, 28007 Madrid, Spain

In this Special Issue, titled “Biofilm-Related Infections in Healthcare”, we have reported considerable progress in understanding the physiology and pathology of biofilms. However, our current diagnostic and therapeutic capabilities lag behind this knowledge, primarily due to a deficit in standardized microbiological tests for identifying biofilm producers and assessing antibiotic susceptibility in these complex bacterial communities.

The inherent metabolic diversity and the shielded microenvironments within biofilms undermine the efficacy of traditional susceptibility assays, often leading to suboptimal clinical decision making and patient outcomes [1,2]. This challenge is further compounded by clinical guidelines that lack the necessary precision for managing biofilm infections effectively, highlighting an urgent need to inspire clinical trials that could foster improved diagnostic and treatment modalities [3]. The refinement of these guidelines should be predicated upon a stringent evaluation of the literature and solid evidence from clinical studies [4].

We advocate for innovative diagnostics and treatment strategies in response to these clinical contingencies. The development and standardization of biofilm susceptibility assays, such as the minimal biofilm eradication concentration (MBEC) and the minimal biofilm inhibitory concentration (MBIC), demand harmonization across research and clinical practice to unlock their full potential [5].

Looking ahead and embracing novel methodologies in biofilm detection and characterization is imperative. Pioneering imaging techniques, cutting-edge molecular diagnostics, and identifying new biomarkers must be expedited, focusing on translating these advances into practical applications within clinical microbiology laboratories. The timely and accurate detection of biofilms is critical in improving therapeutic outcomes, as early interventions are often associated with better patient prognoses. However, the absence of reliable in vivo imaging techniques for biofilm detection presents a significant obstacle in treating these complex infections. Traditional diagnostic systems, such as X-ray imaging and the use of radiolabeled white blood cells, are hampered by the need for invasive sample collection and are prone to failure due to sampling errors, as seen in orthopedic implant-associated infections [6,7]. There exists an imperative need for innovative imaging methods that can non-invasively quantify biofilm presence in real-time. This development would substantially transform the clinical management of biofilm-associated infections. The quest for such a diagnostic tool has led to the exploration of targeted probes for medical imaging capable of specifically detecting bacterial biofilms. Emerging technologies, like the PET tracer 18F-fluorodeoxysorbitol [8], have shown promise in acute infection settings but fall short in addressing the unique challenges posed by chronic biofilm infections. The use of antibiotic-based imaging probes is also being explored [9,10], but these are often limited by their specificity to particular bacterial classes and an unverified ability to infiltrate biofilms and target bacterial cells effectively [11]. More recently, fluorescently labeled peptides have been proposed as promising candidates in biofilm-specific in vivo imaging agents that would improve the diagnosis of several clinical infections [12]. However, a key challenge in this domain is the delivery of adequate amounts of the imaging probe to biofilm sites. This underscores the necessity for groundbreaking imaging probe design and delivery mechanism advancements.

In terms of combatting biofilm, new developments are particularly exciting. Innovative approaches, such as using bacteriophage therapy [13], antimicrobial peptides [14], and the interruption of quorum sensing pathways [15], offer novel means to disrupt and eradicate biofilms. These strategies, combined with traditional antimicrobial therapies, are paving the way for combinatorial treatments that can address the multifaceted challenges presented by biofilms [16].

For implant-related and non-implant-related infections, the future holds the potential for creating anti-adhesive surfaces and materials that resist biofilm formation [17]. Such advances would have profound implications for medical device manufacturing and patient care protocols.

One of the most promising avenues for advancing our understanding of biofilms lies in studying the microbiome’s complex ecosystems, particularly the multispecies and multikingdom interactions that govern chronic conditions [18]. This research holds the potential to yield innovative diagnostic and therapeutic approaches, fundamentally altering the management of biofilm-related infections.

In the collaborative spirit that defines our field, we must ensure that research objectives are intimately aligned with clinical demands. Establishing clear and clinically relevant criteria for biofilm-related infections is paramount, as is the need for integrated efforts among microbiologists, clinicians, and researchers to solidify the clinical support infrastructure.

This Special Issue proves the progress in understanding biofilm-related infections and is a clarion call to action. Through continued innovation and standardization of diagnostic and treatment protocols, we aim to refine and enhance healthcare solutions, ultimately elevating the standard of care for patients grappling with biofilm-associated infections.
